# Possibility of the optimum monitoring and evaluation (M&E) production frontier for risk-informed health governance in disaster-prone districts of West Bengal, India

**DOI:** 10.1186/s41043-024-00632-1

**Published:** 2024-09-17

**Authors:** Moumita Mukherjee, Anuj Batta

**Affiliations:** 1 Institute of International Health, Charité - Universitätsmedizin, Berlin, Germany; 2 Einfach Business Analytics Pvt. Ltd., Kolkata, India; 3 Indian Institute of Technology, New Delhi, Delhi India; 4 Quanolytics, New Delhi, Delhi India

**Keywords:** Monitoring and evaluation, Healthcare, Efficiency, Effectiveness, Disaster risk reduction

## Abstract

An efficient M&E system in public healthcare is crucial for achieving universal health coverage in low- and middle-income countries, especially when the need for service remains unmet due to the exposure of the population to disaster risks and uncertainties. Current research has conducted exploratory and predictive analyses to estimate the determinants of sustainable M&E solutions for ensuring uninterrupted access during and after disasters. The aim was to estimate the efficiency of reaching a higher M&E production frontier via the Cobb‒Douglas model and stochastic frontier model as the basic theoretical and empirical frameworks. The research followed a deductive approach and used a stratified purposive sampling method to collect data from different layers of health and disaster governance in a flood-prone rural setting in the Malda, South 24 Parganas and Purulia districts in West Bengal, India. The present mixed-method study revealed multiple challenges in healthcare seeking during disasters and how a well-structured M&E system can increase system readiness to combat these challenges. The stochastic frontier model estimated the highest M&E frontier producing the most attainable M&E effectiveness through horizontal convergence between departments, enhanced coordination, the availability of frontline health workers at health centers, the adoption of learned innovation and the outsourcing of the evaluation component to external evaluators to improve M&E process quality. Although the study has several limitations, it shows the potential to increase technical and allocative efficiency through building skills in innovative techniques and applying them in process implementation. In the future, research on strategy improvement followed by real-world evidence-based policy advocacy is needed to increase the impact of M&E on access to healthcare services.

**Supplementary Information:**

The online version contains supplementary material available at 10.1186/s41043-024-00632-1.

## Introduction

The normative approach for designing health policy directives evolved gradually in the history of healthcare economics, shifting the focus from free market solutions to altruistic solutions to correct market failures [1]. Despite the availability of effective equitable health schemes, covariate shocks such as natural disasters interrupt equitable access to healthcare, impacting population health outcomes, with greater impacts on the child population [2–5]. Additionally, the impact is manifold in a less-equipped, less resilient supply-side environment, aggravating poor health outcomes [6–8]. One major component of the strengthening health system is focusing on enhancing different elements of any select implementation, such as SMART goal setting and adopting an efficient innovative M&E toolkit [9]. Given this context, this study aims to investigate the determinants of an efficient and effective M&E system to increase the health system’s resilience and render uninterrupted services during disasters. It attempts to explore the role of resource availability, local governance and moderating catalysts to strengthen it to push the existing M&E frontier to a markedly higher but attainable level.

The impact of natural disasters on population and health is evident from UNISDR [7] and World Bank [10] reports. Over a period of two decades, natural disasters affected 4.4 billion people and killed 1.3 billion people, and the world economy incurred a $2 trillion loss. Between 1970 and 2020, natural hazards affected 6.9 billion people and killed more than 2 million people in the Asia–Pacific region, indicating that the impact intensified over time [11]. Among the different influencing factors, systemic gaps in the M&E component of social programmes eventually contribute to increasing the gaps in social achievements, leading to increased economic costs [12]. The direct economic cost of disasters is increasing to $170 billion, indicating significant concerns for policymakers [8].

Among the different types of natural disasters, floods, cyclones or heatwaves affect the health status of children living in coastal areas, riverbank regions or arid regions [4]. These events influence the population directly by increasing morbidity and mortality due to a lack of access to safe water and sanitation and indirectly by prohibiting access to health centers due to inundation or extreme heat [13–15]. An increase in the incidence of common ailments creates pressure on the health system with less readiness even for routine health services, contributing to disaster-led social inequity, which is a greater global health concern [16–20]. Under the changing climate scenario, in 2050, South Asia is expected to have 59.1 million undernourished children under the age of five, which is greater than that in any other developing region [15]. The current study explored ways to improve system readiness through sustainable M&E solutions to reduce the impact of climatic shock-induced health shocks on poor children in the exposed pockets of the three districts in West Bengal, India, and identified the determinants of an effective M&E model for strengthening governance.

Interrupted service availability during disasters increases child-specific health deprivations—mortality and morbidity—and increases vulnerability further [21–25]. Gaps in M&E system effectiveness through improvements in operational efficiency are considered impactful interventions, which are missing in many health system responses to disasters [13, 26]. As reported in the literature, M&E enhances the volume and veracity of program outcomes by identifying the best resource allocation and process improvement methods, which, if replicated in other similar contexts, can contribute to greater social benefits [27, 28]. Monitoring is an M&E component that assesses the process and outputs throughout the implementation period, whereas evaluation measures the degree of outcome achievement at different time points [29]. A lack of understanding about ex ante, ex post and intermittent analyses is responsible for the limited awareness of the importance of the effectiveness of the M&E system as a success accelerator in healthcare programmes. With respect to the efficiency gaps in the M&E component of governance, the World Bank [10] study has shown that ontological gaps in the logical framework, unskilled resource use in program implementation, and irregular periodic evaluation of performance and process are contributors to the failure of the M&E component to ensure effectiveness. Zedtwitz and Gassmann [30] inferred from their research that internationalization of the M&E component can increase the operational efficiency of the M&E system through adoption of innovation, which ultimately increases comparative advantage [31]. One study assessing the process effectiveness of a nutrition programme in Gujarat, India, revealed that 89% of frontline workers lacked orientation training, whereas another study showed that capacity building for Anganwadi workers improved performance quality significantly and contributed to programme coverage [32, 33]. Moreover, M&E performance depends on several internal governance attributes, such as the culture of decision-making, efficiency in resource allocation, availability of skilled health human resources, and social leadership capabilities [34, 35]. One recent study demonstrated the application of novel analytics to predict the progress and trends of certain diseases to control their spread [36]. They proposed a hybrid model combining logistic and susceptible-exposed-infectious-recovered (SEIR) models so that forecasting based on existing trends can be made with the highest accuracy [36]. If such novel and innovative concepts are adopted and analytic-driven M&E is designed, it might exponentially increase M&E effectiveness. Second, in terms of innovative monitoring systems, another study on air quality monitoring revealed that monitoring air quality during lockdowns during pandemics helped to assess how to maintain good air quality even when situations became normal. Furthermore, a study evaluating public health disaster response in North America revealed that learning from previous M&E implementation challenges is a major barrier to improving M&E effectiveness [37]. According to a set of studies, the lack of initiatives in learning from the past lessons of health response programmes during and after a disaster retards the exploration of factors that may improve process efficiency and enhance M&E effectiveness [26, 37–42]. Therefore, a smart monitoring system built on lessons learned from past implementations during a disaster can help create effective policy decisions and maintain efficient service delivery learning from structured innovative monitoring systems adopted during a disaster [26, 37, 42–44].

The stochastic frontier model has been applied in the literature to determine the degree and magnitude of influence of the technical efficiency and quality dimensions of M&E systems with higher reliability and robustness [45]. Additionally, other contributors visible in different studies are the lack of initiatives and related resource allocation aimed at capacity-strengthening initiatives for community health workers [46–48]. Furthermore, studies adopting stochastic frontier analysis or data envelopment analysis have identified significant catalysts responsible for successfully amplifying the impact of enabling factors [49]. Among them, system strategies, along with convergent service delivery linking different line departments, are found to be significant, on the basis of which the M&E theoretical framework under exposure to covariate shocks is sometimes constructed [50]. Given the context of the present research, the stochastic frontier model is applied, and different layers of models are tested to obtain the best model with a higher level of technical efficiency where system factors are considered independent variables.

Notably, different initiatives have started to test how far an integrated M&E system can increase the efficiency of programs to enhance child health outcomes. In this process, different components include capacity building by mid-level practitioners and implementers responsible for delivering child-centric services; designing a risk and impact M&E framework for health governance; and covering preparedness, response and mitigation components in health disaster management plans. This comprehensive plan aims to ensure child-specific healthcare services during disasters and reduce the defaulter rate. In this process, an integrated M&E system must be tested with periodic spatial and temporal analyses of child-specific indicators to inform the system of risk to fill the service delivery gaps. Therefore, a comprehensive situation analysis becomes a prerequisite before developing a context-specific M&E model for designing specific indicators and testing its feasibility, and the current study makes little effort in this direction.

According to the Intergovernmental Panel on Climate Change [51], to reduce the severity, interconnectedness, and irreversibility of the impacts of climate change, risk-informed institutions are necessary to help in the adaptation process, reducing exposure to risk and vulnerability to climate events. Enhanced M&E output adds to the adaptation process, further increasing coping ability and system resilience to combat shocks. Climatic and nonclimatic drivers influence the severity of child-centric health service delivery, affecting supply-side performance and the community's responsiveness to policies and programs [52]. The evidence suggests that community inclusiveness increases in an efficient supply-side environment, ensuring return on investment in child-centric services [52]. Effective management through the production of an ideal quantity of M&E output with quality has become a threshold standard for ensuring the improvement of development indicators in vulnerable geographical pockets [16, 35, 53].

Given this context, the current research objective is to conduct a situation analysis to estimate the risk-informed M&E production frontier with the least inefficiency after identifying the supply-side vulnerabilities and risks in accessing child health services. This objective is to satisfy the goal of increasing the elasticity of service effectiveness by reaching a higher M&E frontier in three of the disaster-prone districts in West Bengal, India.

**Table:** 

The context of India and West Bengal	
• People in different parts of the developing world are living at risk of harmful consequences caused by natural disasters and existing vulnerabilities, reducing the capacity to be resilient [54, 55]. Poverty and social exclusion, challenges in governance, barriers to service delivery with limited preparedness, and suboptimal institutional frameworks without innovation are contributing to major vulnerabilities and hindering the resilience-building process [23, 56, 57] • The number of natural disasters occurring is now four times greater than it was two decades ago [58]. Compared to cyclones or droughts, floods are major disasters that cause the loss of human life, affecting the highest total population, the highest financial loss and the highest percentage of people killed or causing total damage. West Bengal has a long history of natural disasters from 1737 to 2017. Many parts of the state in the last 51 years (1960–2017) have shown evidence of natural hazards such as floods, cyclonic storms, earthquakes, droughts and other disasters • West Bengal is among the most critically disaster-prone states in India. Natural disasters are common phenomena in West Bengal due to their multihazard profile. The southern districts of South 24 Parganas, Kolkata, Howrah, Hoogly and East Midnapore are highly exposed to cyclones. Almost all districts except parts of Bankura and Darjeeling are prone to floods, and the Purulia district suffers from excessive heat waves. Earlier works identify the shocks and stress that sometimes triggered a crisis in these vulnerable districts. Furthermore, compromised system resilience due to greater exposure to floods with a lack of alternative livelihoods and limited institutional capacity requires collaborative effort between the government and civil societies [59]	

Therefore, reducing the social and economic costs of targeting implementation gaps during disasters is crucial [8]. In such circumstances, the potential of strengthening M&E is less realized with suboptimal capabilities, as is the case for other developing counterparts worldwide [9, 60–62]. Exploring the determinants of M&E effectiveness is the step in formulating the pathway of periodic risk and impact analysis to strengthen the M&E system. The three districts in West Bengal State of India considered in this study—Malda, South 24 Parganas and Purulia—are in such a vulnerable pocket; thus, periodic climate risk and impact analyses are needed to ensure service delivery during and after the occurrence of natural hazards. Therefore, exploring the factors affecting M&E effectiveness when designing a framework for periodic climate risk and impact analysis is needed to increase health system readiness.

The Smart Art of Literature Summary.

**Table:** 

Evidence of Health and nutrition programme effectiveness in LMICs	Investigating the effectiveness of strategies to increase efficiency of health and nutrition intervention programme for ensuring access to service during disaster has not done so far, especially in India
Impact of disaster on health and nutrition outcome achievements in India	Though there is increase in coverage with fall in inequity in access to health and nutrition services in India, levels of poor outcomes have not been reduced by adequate magnitude, especially in disaster-prone pockets
Health and nutrition governance—operational and allocative efficiency led effectiveness	Dimension of the effectiveness and efficiency of the M&E component, for example the impact of sharing those data with community—is not conducted especially on the current geophysical setting
Sub-optimal institutional capacity in process effectiveness – frontline workers, fund mobilisation, community focus	Dimension of the effectiveness and efficiency of the M&E component, for example the impact of sharing those data with community—is not conducted especially on the current geophysical setting
Strategic effectiveness towards achieving operational efficiency of M&E	Lack of research is evident on exploring the influence of institutional capabilities and structure in integrated manner on efficiency of M&E system of intervention programme as well as the role of process effectiveness to strengthen that influence further
Stochastic frontier analysis/data envelopment analysis	Need for exploring the factors to strengthen M&E effectiveness – ways of performance measurement, efficiency in identifying and estimating the population in need and succeed in targeting on them, to what extent process effectiveness leads to achieve efficiency, whether gaps in service are measured periodically to identify the children, pregnant women and lactating mothers who are missed out, how far quality is maintained and measured, and steps taken to modify M&E strategies
Themes	Gaps Identified

## Methodology

**Smart art methodology chart** 
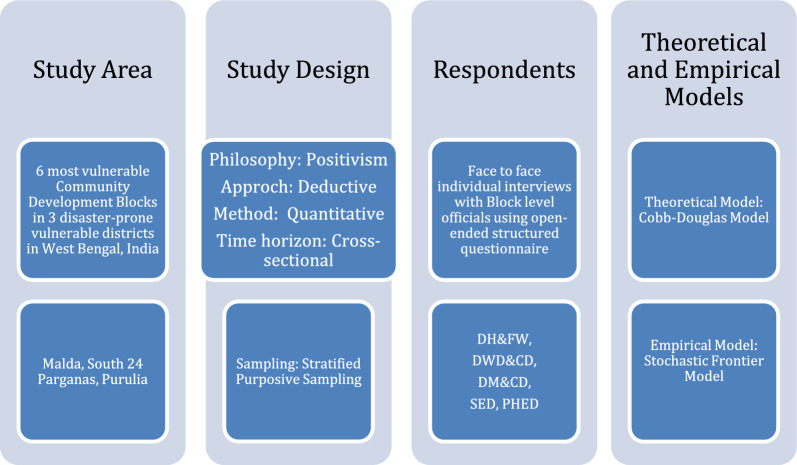


### Study area and population

West Bengal is situated on the east and stretches from the mountains to the sea. West Bengal is divided into five administrative divisions, Burdwan, Jalpaiguri, Presidency, Medinipur and Malda, which are further divided into 23 districts. The region is very distinct from hills to the riverine delta.

The state has a total area of 88,752 square kilometers, is the 14th largest in terms of area, is home to nearly 92 million people and is the 4th most populous state in India. It is the second most densely populated area, with a population density of 1028 people per square kilometer. The population of West Bengal increased from 80.2 million in 2001 to 91.3 million in 2011, accounting for 7.5% of India’s total population. The child population (0–17 years) constitutes 33 percent of the total population (29.9 million), and adolescents (10–19 years) constitute 20 percent (18.2 million). It is predominantly rural in nature, with almost 32% of the urban population being in the 4th highest urbanized state in India. The Schedule Caste (23.5%) and Schedule Tribe (5.8%) are the major socially marginalized groups in the state. The sex ratio in West Bengal between 2001 and 2011 increased from 934 females per 1000 males to 950 females per 1000 males. Although the literacy rate improved from 69.0% in 2001 to 76.3% in 2011, the female literacy rate was lower than the male literacy rate by 11% points [63] (Table 1).

**Table 1:** Demographic profile of the population in 3 districts and the state

District Name	% of female Population	% of Child Population	Child Sex Ratio	% of SC Population	% of ST Population	% of Female Literate	Workforce Participation Rate
Malda	48.7	15.3	960	21.6	8.4	44.6	38.8
South 24 Parganas	48.9	12.6	963	30.2	1.2	71.4	36.3
Purulia	48.9	14.2	953	17.6	19.5	56.5	43.2
West Bengal	48.7	11.6	956	23.5	5.8	70.5	38.1
India	48.5	13.6	918	16.6	8.6	43.1	39.8

Source: Census 2011

The demographic profiles of the population in the study districts are presented along with the state- and country-level figures. The percentage of the female population is almost identical and less than 50% of that of the male population across the districts. A little heterogeneity is evident with respect to the child population; however, the sex ratio depicts a similar pattern of male‒female distribution as that reflected in the adult population. The percentage of the socially marginalized population is much greater in South 24 Parganas and Purulia and is greater than the state average. The female literacy rate is higher for South 24 Parganas. The unemployment rate is very high across districts.

### Data collection

The study involved a desk review of the literature on the current health status of children and their access to healthcare during normal times vis-à-vis disaster time in the district of concern to design the instruments under a mixed-method approach following positivism and critical realism philosophy. A review of the literature was conducted as background research for the main primary study. The research databases searched included ProQuest and Google Scholar with filters for the last five years; public healthcare; journal articles; and the English language. The search terms used were as follows: monitoring and evaluation in healthcare, sustainable healthcare, healthcare for disaster management, healthcare efficiency, healthcare effectiveness, disaster management healthcare, monitoring and evaluation, flood areas, stochastic frontier model, social and economic cost for disaster management, Cobb‒Douglas model, child health, and public healthcare for disaster management. The results indicated that there were slightly more than 400 conference and journal articles in the first stage. However, to maintain the quality of the articles, the search was limited to journal articles, which resulted in 296 journal articles. Among those 296 journal articles, 46 were included because of their relevance to the current research.

The study followed a stratified purposive sampling procedure to create a sample of respondents. The community development blocks are stratified by the degree of vulnerability according to the exposure to disaster, namely, more vulnerable, moderately vulnerable and less vulnerable, by collecting information about the impact on sector-specific indicators. Service providers are purposively selected from the block-level governance of select blocks. The quantitative data are collected via a structured questionnaire, and at the block level, officials who are interviewed face-to-face individually are selected:

- Block Medical Officers, Health (BMOH) from the Department of Health and Family Welfare (DH&FW);
- Child development project officers (CDPOs) and block welfare officers (BWOs) from the Department for Women and Child Development (DWD&CD);
- Block Disaster Management Officers (BDMO) from the Department of Disaster Management and Civil Defense (DM&CD);
- School inspectors (SIs) from the School Education Department (SED) and assistant engineers (AEs) from the Public Health Engineering Department (PHED).

The participants were interviewed to identify programme implementation-related challenges due to the occurrence of hazards to assess the barriers to service delivery at all levels of governance due to floods. In each of the districts, 60 to 75 officials from the selected departments are engaged through departmental convergence to ensure uninterrupted delivery of healthcare services to the children during the disaster. Among them, 10 CDPOs, 10 BDMOs and 10 BMOHs, 10 SIs, and 5 other officials, such as AE, BWO and SBCC workers, were interviewed from each of the 3 districts to assess the research issue, and a total of 112 interviews were successful.

**Smart art: conceptual and analytical framework** 
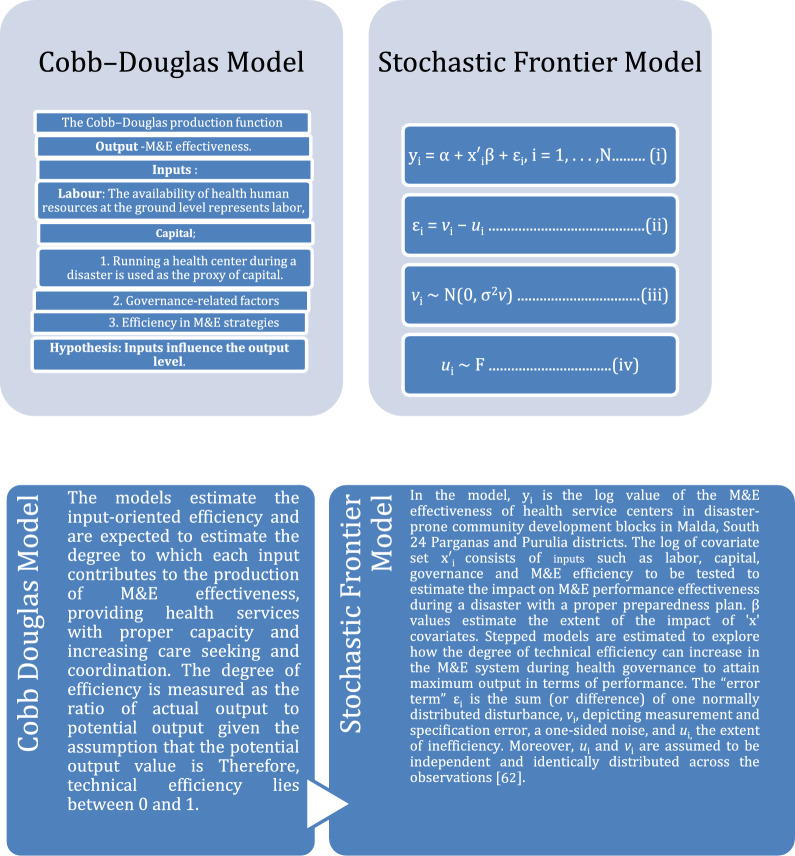


The collected data are preprocessed via Stata 14.0. The data wrangling steps include normalization and missing value handling. The internal consistency between the items used to create the variables is tested via Cronbach’s alpha test on the items constituting the governance variables. The Cronbach’s alpha values revealed that almost all the variables, except Diversity in Connectivity, exhibited greater consistency among the items composing the variable (Table 2). The estimated stochastic frontier models have undergone different postestimation techniques, namely, the likelihood ratio (LR) test, efficiency test (AIC) and consistency test (BIC). STATA 14.0 was used for the econometric analysis of the quantitative data. Bivariate and multivariate analyses were performed to explore the research objectives.

**Table 2:** Cronbach’s alpha test results for the variables used in the analysis

	Cronbach alpha values
Output of M&E system	0.6832
Learning and innovation development	0.8154
Quality of process implementation	0.7821
Learning and adaptation	0.7011
Convergence in decentralized system	0.8802
Diversity in connectivity	0.5160
Outsourcing M&E component	0.8044

The study contributes to the strategic management of implementation programmes at the block level of governance in 3 select disaster-prone districts in West Bengal, India. The findings will help to build and maintain an integrated M&E system to ensure child-centric health and nutrition services during disasters. It helps block-level governance identify how far vertical and horizontal departmental integration, learning and adaptation of innovation and, to some extent, timely outsourcing of select M&E components are the determinants of a successful and effective M&E system to run uninterrupted services. The goal is to reduce child mortality, malnutrition and morbidities in vulnerable populations.

## Results

### Context of child health outcomes—associated risks due to gaps in healthcare access in West Bengal and in the study districts

According to the findings of the National Family Health Survey 5 (IIPS, [2]), the percentage of respondents who had four or more ANC visits was 75.8%, which is higher than the state average in Malda and South 24 Parganas and profoundly low in Purulia. Moreover, most of them visit from the first trimester (72.6%), indicating improvement in coverage of full antenatal care services in the state, where similar patterns, such as full ANC check-ups, are visible across the study districts (higher than the state average in Malda and South 24 Parganas and lower in Purulia). In contrast, PNC check-ups are lower in Malda, such as in the Purulia district, than in the state average. The percentage of women who had a live birth in the five years preceding the survey and who received a postnatal check within two days of birth for their most recent birth was 68.0%. A total of 91.7 percent of deliveries in a health facility cover both private and public facilities, and approximately 3 out of 4 institutional deliveries have taken place in public facilities. Although the rate of institutional delivery was greater at the state level than at the district level, the opposite scenario is evident with respect to delivery in public facilities, which was surprisingly greater in Purulia. The percentage delivered with assistance from health personnel was 94.1%, which was lower in Malda than in the state and other district averages under study, revealing similar patterns, such as PNC uptake—reflecting a shortage of skilled health workers leading to less access to their service in terms of availability, which demands further research. Among women who had a live birth in the 5 years preceding the survey for the most recent birth that was delivered in a public health facility, the average out-of-pocket expenditure per delivery in a public health facility was Rs. 2683 and is comparatively lower in Malda district. The percentage of children (12–23 months) who were fully vaccinated before the survey (according to a vaccination card or the mother's recall) was 87.8%. The percentage who received the most vaccinations in a public health facility was 96.3% in the state. Immunization uptake was greater in South 24 Parganas than in the state average (Table 3).

**Table 3:**
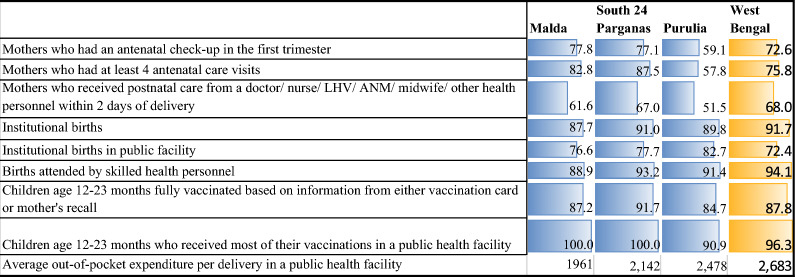
Status of maternal and child health service access in the state and the study districts *Source*: IIPS (2021) [2]

### Findings from the primary study

The distribution of respondents in the three study districts revealed the highest degree of participation in Purulia, a comparatively moderate degree of participation in Malda and the lowest degree of participation in South 24 Parganas.

**Fig. 1:**
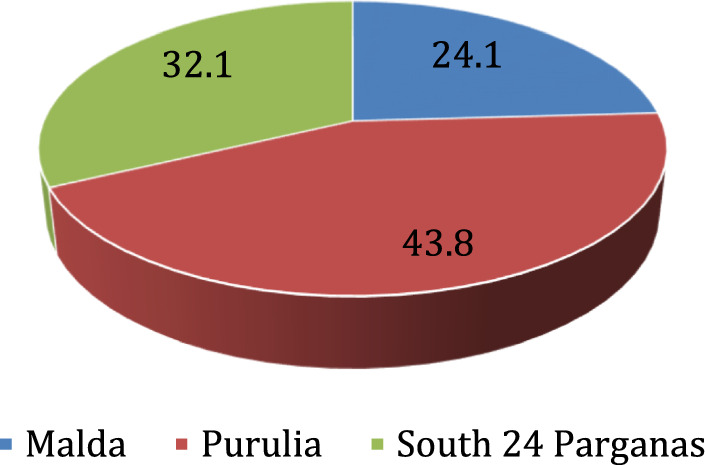
Distribution of the respondents in the three study districts

**Fig. 2:**
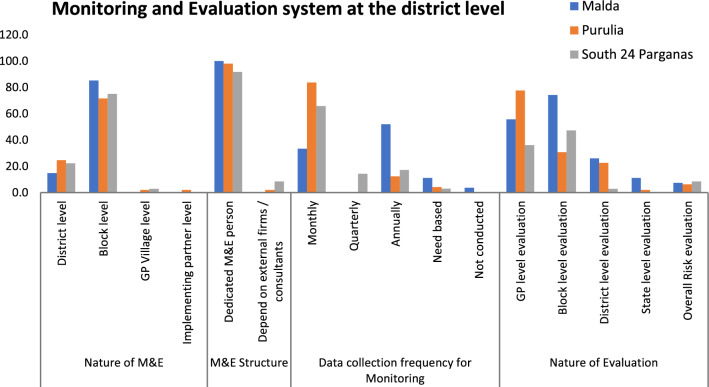
District-level monitoring and evaluation system prevailing at the time of the study

This study gathered information on the existing M&E structure at the governance level, the frequency of data collection and analysis for monitoring, and the nature of the evaluation conducted. Evidently, M&E activities are mostly the responsibility of block-level officials appointed as M&E personnel. According to the interviewed block representatives of governance, monthly monitoring is practiced by most of them in Purulia (83.7%), 65.7% of them in South 24 Parganas, whereas only 33.3% of the block representatives interviewed in Malda reported the practice of monthly monitoring activity. Among the representatives of Malda, more than 50% reported that they practice annual monitoring of routine services. In contrast, evaluation reflects the joint participation of gram panchayat and block-level officials, with district-specific variation in the degree of responsibility, as reflected in the study (Figs. 1 and 2).

### Results from the stochastic frontier models (Table 4)

**Table 4:** Findings from stochastic frontier models

	Model 1	Model 2	Model 3	Model 4
Availability of ASHA, ANM	1.71**	1.68**	0.81	0.79
The operational bottleneck in running the health center due to the impact of the flood on the stock of medicines, equipment	0.05***	0.02	0.01	0.01
Convergence in decentralized system	0.28***	0.03	− 0.08	− 0.10
Diversity in connectivity	0.32	0.21	0.08	0.05
Learning and adaptation		0.47***	0.00	0.00
Learning and innovation development		0.16***	0.10	0.10
Quality in process implementation		0.02	0.00	0.00
Adoption of learnt innovation in M&E to improve process quality			0.03***	0.02***
M&E outsourcing				0.15**
Technical Efficiency	**0.136**	**0.160**	**0.944**	**0.946**
Wald chi2	31.590	108.824	141.271	153.058
p Value	0.000	0.000	0.000	0.000
N	112	112	112	112
AIC	652.8	608.7	603.6	600.5
BIC	671.8	635.9	633.5	633.2

The value of efficiency levels are in bold to show the efficiency estimates which are crucial outcomes of stochastic frontier models

*p < 0.1, **p < 0.05, ***p < 0.01

The LR test results assume that Model 1 is nested in Model 2, Model 2 is nested in Model 3 and Model 3 is nested in Model 4. The *P* values revealed that Model 2 was better than Model 1, Model 3 was better than Model 2, and Model 4 was the best model among the four estimated models (Table 5). AIC tests the efficiency and has shown that Model 4 is the best finite-dimensional model with the assumption that the true (unknown) model has infinite dimensions. On the other hand, according to the results of the BIC tests of consistency, where the true model is finite, the best correct model is Model 4, which satisfies the condition that the probability of achieving technical efficiency toward the value ‘1’ increases with increasing population size.

**Table 5:** Likelihood ratio tests

	(Assumption: Model1 nested in Model2, Model2 nested in Model3, Model3 nested in Model4)		
	Model 1 and Model 2	Model 2 and Model 3	Model 3 and Model 4
Likelihood ratio test (chi2)	50.05	7.09	5.09
Prob > chi2	0.0000	0.0077	0.0240

Therefore, all the tests show that Model 4 is the best model and produces the highest level of technical efficiency. Next, the firm's degree of returns to scale in the production process is tested. Therefore, deviation from the use of constant returns to scale is not significantly different from zero (Table 6).

**Table 6:** Test of the firm's deployment of constant returns to scale in the M&E production process

	Estimated coefficient (95% CI)	*P* Value
Model 1	0.763 (− 0.823 to 2.349)	0.346
Model 2	0.703 (− 0.636 to 2.042)	0.304
Model 3	− 0.176 (− 1.383 to 1.032)	0.776
Model 4	− 0.203 (− 1.383 to 0.978)	0.736

Figure 1A shows the distributional pattern of the predicted technical efficiency of Model 1 and Model 2, and Fig. 1B shows that for Model 3 and Model 4. It is evident that the distributions of predicted technical efficiency are right skewed in Model 1 and Model 2, which are corrected after the implementation of learning and innovation (Model 3) and partial outsourcing in the M&E process (Model 4).

In **Model 1,** four inputs are incorporated—health human resources, availability of medical stock and equipment, and degree of horizontal and vertical integration within the healthcare department and other line departments—to determine whether they have any significant influence on M&E performance effectiveness during or after a disaster. Model 1 shows that the availability of ANM and ASHA in health centers, the condition of the health centers due to flood exposure, and the degree of convergence between departments affect the effectiveness of the M&E system in generating smooth health care access during a disaster. As reflected in the model, the availability of ASHA and ANM in the center during a disaster and constrained working conditions in the center increase the need for integrated M&E coordination to ensure proper preparedness analysis and planning, depicting significantly greater M&E effectiveness at the 95% and 99% levels of significance, respectively. The model also reflects that ‘excellent to satisfactory’ levels of horizontal convergence in M&E activities with a team of health workers and integrated coordination between health and disaster management to prepare a joint health action plan for disaster-prone GPs (to implement in pre, during- and postdisaster situations adopting scientific risk and impact analysis techniques) significantly increase the effectiveness of the M&E system—at the 99% level of significance, with a moderate magnitude of influence on the average effectiveness. However, the influence of diversity and connectivity was not significant. The combined technical and allocative efficiency estimated from this model is **0.136**.

In **Model 2**, variables representing the moderating factor set ‘efficiency of M&E strategies’ comprising ‘Learning and Innovation Development’, ‘Quality of Implementation Process', and 'Learning and Adaptation’ are included to test the second hypothesis. The capacity development of frontline workers with innovative approaches in data collection, maintenance, analysis and planning helps to sustain the contribution of the labor component significantly at the 95% level of significance. Moreover, learning and adopting the innovative M&E approach significantly increases M&E effectiveness at the 99% level. Model 2 shows that both governance factors positively but not significantly affect M&E effectiveness; the direct influence of horizontal convergence and vertical integration becomes weaker. Therefore, departmental convergence and integrated coordination affect M&E performance effectiveness through efficient M&E strategies. This is reflected through integrated learning and adaptation and shows that the combined efficiency increases from **0.136** to **0.160**.

The results improve further in **Model 3,** where the interaction between the adoption of learned innovation and process quality improvement is included. In other words, technical improvement in the process of risk and impact analysis, e.g., with the learning and application of analytics, is likely to strengthen the usability of the M&E system. This model shows that the significance levels of all the other model factors are confounded by the inclusion of innovative risk and impact assessment processes—more specifically, they are based on real-time evidence. It can be inferred that the impact of disasters on operational efficiency gradually weakens. This model reflects a sharp increase in efficiency from **0.160** to **0.944**.

**Fig. 3:**
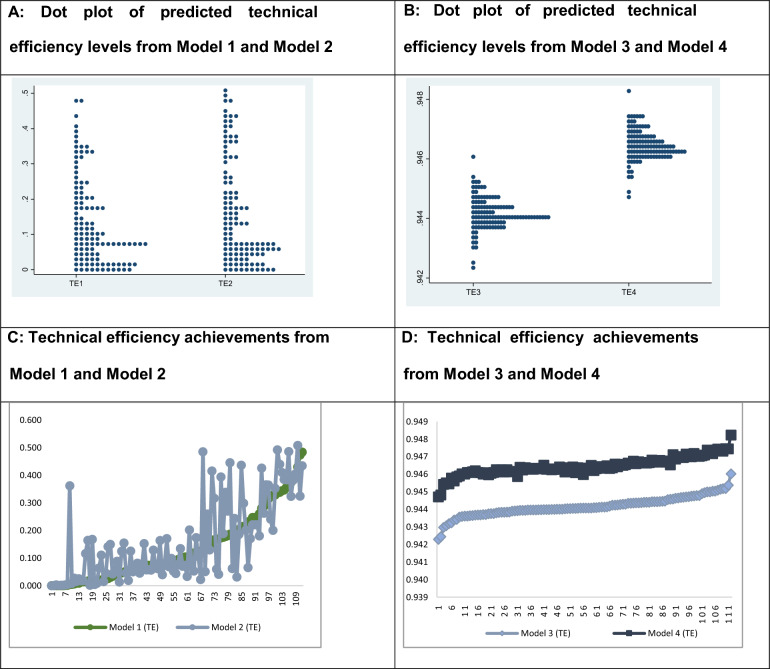
Technical efficiency charts

To increase efficiency further (**Model 4),** this model is tested by incorporating a new variable, outsourcing of some of the M&E components, for example, ex ante and ex post evaluations, to remove M&E operational bias and improve effectiveness. Therefore, if the implementation of periodic health risk and impact assessment is conducted with quality improvement in the M&E process and outsourcing the evaluation component to external evaluators significantly influences M&E effectiveness at the 95% level of significance, the predicted efficiency level increases from **0.944** to **0.946 (**marginally**)** (Fig. 3).

In summary, the present research explored how to improve the M&E system of health service delivery in disaster-prone vulnerable pockets by adopting a convergence mode with disaster management and different line departments to ensure child-centric services. This study revealed that governance-related factors influence the achievement of the highest M&E production possibility frontier. The adoption of innovation is key to success, along with the outsourcing of certain components. It also tested ways to improve the technical and allocative efficiency of the comprehensive M&E system to push it to the highest effectiveness frontier, identifying the best possible combination of inputs. This study contributes to the theory of the M&E effectiveness strategy by applying the foundational theory of the neoclassical production function to combat system inefficiencies during any disaster. The conceptualization of a present-day implementation research problem from a neoclassical theoretical lens strengthened its root. This helps to compute robust results when a stochastic model is applied for empirical analysis. This could be termed a novel contribution of the present study, although it is small and indicative given the limited geographical focus. Future research should test this strategic model in different geographic settings as well as in different disaster contexts, such as M&E, for surveillance and pandemic management.

## Discussion

The current study tested how far systemic factors affect the comprehensive M&E production possibility frontier and how the levels of combined technical and allocative efficiency of an integrated M&E system can be enhanced so that the health system can reach maximum effectiveness in terms of the frontier. The stochastic frontier model thus estimated the best possible input combinations to achieve the maximum possible effectiveness (output) in controlling the impact of the disaster on health service delivery. The geographical setting selected for the primary study was three disaster-prone areas in West Bengal on the basis of the relatively high degree of exposure to risk and vulnerabilities.

To ensure health service delivery during a disaster, the Disaster Management and Civil Defense departments, which are equipped with technical assistance from an international organization, have initiated the process of developing an integrated M&E system where the selected districts are chosen as the implementation settings [65–67]. It comprises components to assess the risks and vulnerabilities in access, the status of service delivery gaps under exposure to risk, and how it impacts child health outcomes. The initiative aims to reduce child-specific health vulnerabilities, adjusting system risk through increasing system resilience. The current study examined how system resilience can be ensured through increasing technical efficiency in the allocation and utilization of system resources with efficient M&E strategies (integrated departmental coordination, action, capacity building and implementation). The study tested how much the initiatives help increase M&E effectiveness, measured in terms of reaching the highest M&E production possibility frontier (when technical efficiency → 1, i.e., the maximum), assuming that higher M&E performance (production) increases the resilience of the system and adjusts it to disaster risk to ensure child-specific services. The present study is a valuable addition to this initiative from the academic side, as it focuses only on the health sector.

The results of the stochastic frontier model have led to different policy dimensions. First, **Model 1** shows that the implementation of integrated M&E in convergence mode has greater efficiency in terms of performing effectively. This study is in line with studies that investigated how far a nutrition programme can be successful at achieving horizontal integration with health and education [5, 68–70]. Hawkes et al. [71] mentioned that these opportunities are not yet optimally utilized; therefore, the current contribution adds further. Another study exploring the impact of horizontal and vertical system coordination on the efficiency of the health system in Kenya has shown that challenges in integrated coordination increase transaction cost-reducing efficiency, which decreases the effectiveness of the health system [72]. Another study has shown that inefficient management strategies during the COVID-19 pandemic resulted in delayed health system responses, affecting health services in terms of delayed care, e.g., orthopedic and neurological surgeries in government hospitals in the West Bank of the Palestinian territories [73]. Therefore, if health system resilience is not built by correcting the weaker components of the system, service delivery can be jeopardized during any disaster.

Second, **Model 2** shows that components of M&E strategic efficiency significantly facilitate sustaining the impact of the enabling input of the M&E effectiveness production function through reducing diversity elements and enhancing the connectivity elements in vertical and horizontal integration. The significance of horizontal convergence, connectivity and vertical coordination influencing M&E performance via innovation in M&E capacity building for frontline workers may be due to integrated preparedness planning at the GP level in disaster-prone areas. If it can be added to the district-level platform involving other concerned-line departments connected to child-centric healthcare services, the effectiveness of the intervention will increase, as found in other programmes [23, 65–67, 74].

The empirical model also reflects that vertical integration in governance is highly important for increasing M&E effectiveness, which is in line with the findings of a study in which the monitoring of frontline workers in Gujarat, India, was not successful at achieving results due to a lack of vertical integration in governance, as it created a less efficient frontline system [32, 75]. However, the current study has found a solution to create effectiveness through the adoption of innovations, such as the application of analytics, to improve database management by integrating all the vertical levels, which is visible in two other studies. One study conducted in Uganda showed that improving the health system's capacity innovatively by altering and strengthening resources at integrated and connected local service delivery points increased health system performance in a sustainable manner, which is evident from the findings of the current research [62]. Another study in Nigeria exploring the impact of capacity building of health workers on program effectiveness has shown that contextually customized training materials, guided supervision, innovation in data collection and validation methods using comparable monitoring indicators improved the performance of state malaria programs under vertical integration [76].

Another study in Uganda showed that one of the most underutilized components in health systems management is the proper use of health records and that a mismatch between frontline workers’ and policy-level willingness to build technical capacities is a significant determinant of less utilization; however, such mismatch was not significantly evident in our study [61]. This indicates that the presence of system- and policy-level willingness is the primary criterion for increasing data utilization. In the absence of such information, the initiative for adopting innovation in building efficient M&E strategies will be difficult to initiate. A study in the KSA on health system transformation showed that knowledge building at the implementation level should be combined with research plans and efforts to build strong research governance, which was not tested in the current study and should be focused on the next level of research after the initiation of implementation [77].

However, studies exploring the usability of a strong M&E system as an innovative governance tool show that an integrated M&E system comprising the collection and analysis of bottom-up data and good coordination among policymakers, stakeholders and service providers foster need-driven decisions and policies that ultimately reduce the likelihood of market failure—as evident from a study that explored the capability of a monitoring system to monitor the health workforce in the German Federal State of Rhineland-Palatinate and matches the inference of the current study [78]. Therefore, it can be inferred that the success of an M&E system depends not only on the willingness of the service provider or policy maker but also on the need to integrate crucial stakeholders in the whole process, starting from the planning phase to the implementation phase, to increase its effectiveness. The current study has shown similar findings—M&E effectiveness increases with inter- and intradepartmental integration—and when innovation is adopted, health governance factors use innovation as an instrument to increase M&E effectiveness.

It is apparent that in **Model 3,** the incorporation of one interaction term has improved the predictability and explanatory power of the stochastic frontier model between learning and adaptation and process quality innovation. We examined whether the integrated health work force was trained with innovative technology to improve the quality of the data collection, analysis and use of data integrating the coordination between the health department and disaster management department. The results showed that the quality of the periodic child risk and impact assessment could be improved. In line with the current research, a systematic literature review exploring the barriers to and facilitators of implementing trauma-informed healthcare has shown that the perceived significance of the initiative for policymakers and implementers, flexible policy and training merged with the process of aligning changes, and user feedback analysis are the main enablers [34]. Several studies have identified the importance of the lessons learned to create efficient M&E systems and achieve effective public health responses during disasters [26, 37, 41]. In line with these studies, the current research has shown that improving the effectiveness is possible when learning is deployed in the process. Although the current study included implementers at the ground level, state-level policymakers are involved in the next stage of exploration.

Furthermore, according to Model 3, if the health workforce is trained on innovative concepts during capacity-building activities, the quality of implementation improves through learning, adaptation and application. Concerning the use of innovation, one mixed methods study in England assessing the fidelity of a digital health service programme to the structure specification has shown that variation in the delivery of the digital diabetes prevention programme by four different providers may influence the effectiveness of the process, which was continuously improved on the basis of user experience feedback [79]. Two recent studies have shown the relevance of adopting innovative monitoring systems and applying big data analytics [36, 43, 44]. They have shown how such innovation contributes to more effective informed policy decisions with higher accuracy and minimum error [36, 43, 44]. Therefore, further research is needed to assess how training on innovation will be procured and how fidelity to the requirements of health governance will be created to ensure an appropriate threshold of effectiveness. Thus, periodic risk and impact analysis becomes highly effective at ensuring access to services during disasters embedded in horizontal and vertical integration between and within departments.

**Model 4** incorporates one variable representing the views of different stakeholders on M&E outsourcing to increase M&E effectiveness. According to the respondents, outsourcing M&E activities to external evaluators or agencies significantly increases the effectiveness of the intervention. The literature reflects differing views of outsourcing when a sector faces any disruption—whether technological, social, or natural [10]. In such circumstances, that sector designs short-term plans to resume and long-term plans for recovery to maintain service continuity sustainably [10, 62, 80–82]. Studies by Aragão and Fontana [80] explored the policy inclination to disfavour outsourcing during disruptions such as natural disasters and inferred through their study that the efficient use of outsourcing increases the likelihood of service continuity during such disruptions. However, legal and procedural factors should be investigated further to understand the comprehensive set of enablers and barriers to outsourcing affecting service continuity.

M&E skill building significantly increases operational efficiency and process quality, influencing M&E effectiveness under departmental convergence. The integrated M&E with the disaster management department jumps to a significantly higher frontier with the training and adoption of innovations. Finally, outsourcing the evaluation component can further enhance the efficiency level to reach a higher level of M&E frontiers given contextual adversities.

### Study limitations

This study has several limitations. The study was limited by its very small sample size due to time, cost and mobility constraints. Second, the study followed purposive sampling to select the interviewees. However, when the study was conducted, the concept of integrated M&E was piloted in select blocks, which is one of the reasons for purposive selection. In the future, a large-scale longitudinal study should be designed to conduct a large survey covering multiple districts following a stratified random sampling procedure. Second, the analysis uses only classical approaches. No analysis has been conducted to propose a comprehensive decision support system using machine learning/deep learning algorithms. Therefore, it might lack higher predictive accuracy and precision. Future research is required to consider this dimension.

## Conclusion

The present work adds value to the literature in many ways. These findings provide a direction for strengthening the decision support system (DSS) of integrated local governance and identifying the contextual determinants. This study fills such a knowledge gap for any social programme as visible in the literature [32, 45, 75]. Furthermore, to make the system more effective and accessible, each program can design an M&E DSS automating the whole process, starting from data acquisition to analytics and evaluation while considering the determinants related to the data and performing real-time analysis. This topic is under consideration for future research.

The proposed integrated process can be utilized to form a workforce team at the gram panchayat, block and district levels, and the comprehensive fully proofing M&E system can eventually be realized as a pilot. Furthermore, the collection of real-time data for developing preparedness plans can assure health service delivery during a disaster. Two connected real-time databases need to be trained via machine learning and deep learning techniques to modify the action plan regularly and guide the health workforce in disaster-prone areas. Lessons from the implementation in the form of impact evaluation are then documented and applied for further modifications and changes if needed. In the next phase, the tested model can be replicated in other similar vulnerable locations with continuous process improvements based on the user experience, ultimately reducing the impact of disasters on the health outcomes of vulnerable children.

Moreover, the development and maintenance of electronic risk and impact analysis of healthcare services in disaster-prone districts are planned after the development of technical skills among ground-level workers while setting an M&E technical hierarchy at each level of governance on the basis of learning. Continuous research at the implementation level is required to establish, test, implement and run the process cyclically so that the predicted level of M&E output can be achieved after ensuring the predicted level of technical efficiency. Further exploration is recommended to test how to minimize technical inefficiencies via the use of digital health tools to increase social benefits by reducing the cost of intervention.

## Supplementary Information

Additional file1

## Data Availability

This information has been added as supplementary material.

## Appendix

### Variable description

The variables used for testing the hypotheses via the stochastic frontier model are the availability of ASHA and ANM in the center during a disaster; the running conditions of the center during a disaster, which are based on the impact on the availability and condition of the medicine stock; the equipment, water supply, and sanitation in the center during and after a disaster; the degree of departmental convergence in decentralized governance to create a risk-informed M&E plan; the vertical integration adopted in building M&E strategies within a department for the poor and vulnerable population; and the strategic efficiency (moderating factor), which includes the technical efficiency of the strategy used—Learning and Innovation Development, Learning and Adaptation and Quality of Implementation process. The dependent variable was M&E production in the healthcare system.
