# Supplementary material for: Possibility of the optimum monitoring and evaluation (M&E) production frontier for risk-informed health governance in disaster-prone districts of West Bengal, India
Source: J Health Popul Nutr. 2024 Sep 17;43:148. doi: 10.1186/s41043-024-00632-1 (PMC11409740; doi:10.1186/s41043-024-00632-1)
Supplement: Supplementary file 1 — Additional file1 [file 41043_2024_632_MOESM1_ESM.docx]

**Supplementary material**

1. **Thematic matrix of research works reviewed**

| **Themes** | **Gaps emerged** | **Literature** |
| --- | --- | --- |
| Evidence of Health and nutrition programme effectiveness in LMICs | Investigating the effectiveness of strategies to increase efficiency of health and nutrition intervention programme for ensuring access to service during disaster has not done so far, especially in India. | 1. Popkin et al. 2019 2. Bhutta et al. 2013 3. Shekar et al. 2017 4. Alderman et al. 2017 5. Chakrabarti et al. 2019 6. Martinez-Schiferl 2012 7. Leroy et al. 2008 8. Keweloh 2015 9. UNICEF 2018 |
| Impact of disaster on health and nutrition outcome achievements in India | Though there is increase in coverage with fall in inequity in access to health and nutrition services in India, levels of poor outcomes have not been reduced by adequate magnitude, especially in disaster-prone pockets. | 1. Gragnolati et al. 2006 2. Rao and Kaul 2018 3. Menon et al. 2016 4. Chakrabarti et al. 2019 |
| Health and nutrition governance – operational and allocative efficiency led effectiveness | Dimension of the effectiveness and efficiency of the M&E component, for example the impact of sharing those data with community – is not conducted especially on the current geophysical setting | 1. Engle et al. 2007 2. United Nations 2019 3. Knowler et al. 2005 4. Onis 2017 5. Mark et al. 2018 6. Chudasama et al. 2016 7. Chudasama et al.2013 8. Saxena et al. 2015 |
| Sub-optimal institutional capacity in process effectiveness – frontline workers, fund mobilisation, community focus | Lack of research is evident on exploring the influence of institutional capabilities and structure in integrated manner on efficiency of M&E system of intervention programme as well as the role of process effectiveness to strengthen that influence further | 1. Gragnolati et al. 2006 2. Gadhkar et al. 2006; NIPCCD 2004; NIPCCD 2005 3. UNICEF 2018 4. UNICEF GRIP 2016 5. CRIA 2018 |
| Strategic effectiveness towards achieving operational efficiency of M&E | Need for exploring the factors to strengthen M&E effectiveness – ways of performance measurement, efficiency in identifying and estimating the population in need and succeed in targeting on them, to what extent process effectiveness leads to achieve efficiency, whether gaps in service are measured periodically to identify the children, pregnant women and lactating mothers who are missed out, how far quality is maintained and measured, and steps taken to modify M&E strategies | 1. World Bank study (2013 2. Zedtwitz and Gassmann 2002 3. de Meyer and Mizushima 1989 4. Datta et al. 2010 5. Balasubramanian and Raviindran2012 6. Chakrabarti et al. 2019 7. Hawkes et al. 2019 8. WHO 2013 9. Kanjilal et al. 2010 10. IIPS 2017 11. UNICEF 2016 12. Maity 2016 13. Desai and Dubey 2012 14. Nisbett and Barnett 2012 |
| Stochastic Frontier Analysis / Data Envelopment Analysis | Gaps in deployment of SFA model to investigate the best model allowing higher level of technical efficiency considering systemic factors – Learning and Adaptation, Convergence in Decentralised Systems, Vertical and Horizontal Integration, effectiveness of strategies and M&E outsourcing – as covariates | 1. Walburg 2006, 2. Klein 2010, 3. Politt et al. 2010 4. Kollberg and Elg 2011 5. Viteziḉ et al. 2019 6. Bourne et al. 2018 7. Waldman 1994, 8. Bititei et al. 1997, 9. Radnor and Barnes 2007 10. Kolberg and Elg 2011, 11. Yuen and Ng 2012 12. Mesabbah and Arisha (2015 13. Kaplan and Norton 1996 14. Carrick et al. (2013 15. Drumm 2011 16. HSE 2014 17. Lobo and Couto (2016 18. Graham et al. (2003 19. Jain et al. 2008, Tsai et al. 2015 20. Sayeed et al. 2017 21. Parker 2000 22. Hollingsworth 2008, 23. Asandului et al. 2014 24. IDSK 2017, 25. Lodge 2014, 26. Lunn 2014 27. Greve and Hodge 2013, 28. Liu et al. 2014, 29. Hoque 2014, 30. Micheli and Mura 2017, 31. Bourne et al. 2018 |

1. **Methodology Chart**
2. **Construction of governance related factors**

|  | **Learning and Innovation Development** |
| --- | --- |
| 1 | Arranged training for Supervisors/school teachers/ANM/ASHA/Civil volunteers/VLCPC members/AWWs with confirmed participation in adequate number of trainings as per guideline |
| 2 | Trainings helped to conduct monitoring activities in convergent mode |
| 3 | Departmental convergence reduced operational cost for training and capacity building |
| 4 | Supervisors/School teachers/ANM/ASHA/Civil volunteers/VLCPC members/AWWs in disaster-prone areas attended trainings and implemented the knowledge |
| 5 | Meeting with frontline workers conducted after training session to receive feedback |
| 6 | Review meeting with frontline workers help them to know the spatial and temporal analyses |
| 7 | Innovative methods taught and used in data collection and analysis to reduce time of operation |
| 8 | Taught using monitoring results to modify targets and strategies |
|  | **Quality of Process Implementation** |
| 1 | Impact of programme components are measured periodically |
| 2 | Compliance is followed |
| 3 | Data on daily activity are shared with district level after analysis |
| 4 | Monitoring led to design of action plans |
| 5 | Results are shared with Communities and other stakeholders |
| 6 | Follow-up meetings after disaster developed preparedness and response plan |
| 7 | Follow-up meetings after disaster developed recovery and mitigation plan |
| 8 | Monitoring activities conducted during disaster |
| 9 | Data collected on children and women who failed to take service during disaster |
| 10 | Sufficient financial resource for M&E is ensured after first year of implementation |
| 11 | Ensuring that staffs at ground level are performing monitoring in regular manner |
|  | **Learning and Adaptation** |
| 1 | Conduct evaluation of staff performance after disaster |
| 2 | Measured coverage of population after disaster |
| 3 | Risk analysis process followed |
| 4 | Process followed to identify excluded needy children and women |
| 5 | Cost benefit analysis followed in risk analysis to mitigate impact of financial risk |
| 6 | Collection of periodic data on disaster impact analysis |
| 7 | Identification and removal of non-working methods in the M&E to improve technical efficiency |
| 8 | Estimated results are validated at field |
|  | **Convergence in decentralized system** |
| 1 | Convergence established for risk and impact analysis |
| 2 | Coordination steps followed to ensure timely completion of analysis and sharing of results |
| 3 | Capacity building organized in convergent mode |
| 4 | Developed integrated Preparedness plan |
| 5 | Designed and measured integrated performance indicators |
| 6 | Integrated action plan components with disaster management |
| 7 | Risk and impact analysis is tagged with GIS to improve performance |
|  | **Diversity and Connectivity – Vertical and horizontal integration of implementation after risk and impact analysis** |
| 1 | Under vertical integration process mid-level workers act as liaison |
| 2 | Block officer directly sends instructions to common frontline team of response |
| 3 | Frontline workers from each line department worked as members in common M&E team |
| 4 | Alterations done in training modules based on feedback |
| 5 | Frontline workers change pattern of process based on M&E results |
|  | **M&E Restructuring and Outsourcing** |

| 1 | Outsourcing of M&E activities will improve the programme efficiency |
| --- | --- |
| 2 | Saves cost in terms of in-house establishment |
| 3 | Saves cost in terms of human resource |
| 4 | Saves cost for training and capacity building of programme personnel |
| 5 | Increases technical efficiency with deployment of technical knowledge of external experts |
| 6 | Saves time of programme personnel |
| 7 | Increases technical efficiency through improving quality of work in less time |
| 8 | Collaboration increases access to other department’s data |
| 9 | Increases managerial involvement to ensure Planning |
| 10 | Increases managerial involvement to ensure operational efficiency |

|  | ***Output*** |
| --- | --- |
| 1 | Success of Lessons learnt |
| 2 | Success in overcoming Challenges |
| 3 | Increase in coverage |
| 4 | Supply evidence for district level governance |
| 5 | Operational system related practice change after disaster |
| 6 | Detailed documentation through comprehensive report |
| 7 | Dissemination and discussion |

1. **SMART ART: Conceptual and Analytical Framework**
